# Epidural combined optical and electrical stimulation induces high-specificity activation of target muscles in spinal cord injured rats

**DOI:** 10.3389/fnins.2023.1282558

**Published:** 2023-11-10

**Authors:** Xiao-Jun Guo, Ziyi Zhao, Jia-Qi Chang, Le-Wei He, Wen-Nan Su, Ting Feng, Can Zhao, Meng Xu, Jia-Sheng Rao

**Affiliations:** ^1^Beijing Key Laboratory for Biomaterials and Neural Regeneration, Beijing Advanced Innovation Center for Biomedical Engineering, School of Biological Science and Medical Engineering, Beihang University, Beijing, China; ^2^Department of Orthopedics, The First Medical Center of PLA General Hospital, Beijing, China; ^3^Smart Fluid Equipment and Manufacture Lab, School of Automation Science and Electrical Engineering, Beihang Univeristy, Beijing, China; ^4^Institute of Rehabilitation Engineering, China Rehabilitation Science Institute, Beijing, China

**Keywords:** spinal cord injury, epidural electrical stimulation, near-infrared nerve stimulation, muscle activity, neuromodulation, motor function, rehabilitation

## Abstract

**Introduction:**

Epidural electrical stimulation (EES) has been shown to improve motor dysfunction after spinal cord injury (SCI) by activating residual locomotor neural networks. However, the stimulation current often spreads excessively, leading to activation of non-target muscles and reducing the accuracy of stimulation regulation.

**Objectives:**

Near-infrared nerve stimulation (nINS) was combined with EES to explore its regulatory effect on lower limb muscle activity in spinal-cord-transected rats.

**Methods:**

In this study, stimulation electrodes were implanted into the rats’ L3–L6 spinal cord segment with T8 cord transected. Firstly, a series of EES parameters (0.2–0.6 mA and 20–60 Hz) were tested to determine those that specifically regulate the tibialis anterior (TA) and medial gastrocnemius (MG). Subsequently, to determine the effect of combined optical and electrical stimulation, near-infrared laser with a wavelength of 808 nm was used to irradiate the L3–L6 spinal cord segment while EES was performed. The amplitude of electromyography (EMG), the specific activation intensity of the target muscle, and the minimum stimulus current intensity to induce joint movement (motor threshold) under a series of optical stimulation parameters (power: 0.0–2.0 W; pulse width: 0–10 ms) were investigated and analyzed.

**Results:**

EES stimulation with 40 Hz at the L3 and L6 spinal cord segments specifically activated TA and MG, respectively. High stimulation intensity (>2 × motor threshold) activated non-target muscles, while low stimulation frequency (<20 Hz) produced intermittent contraction. Compared to electrical stimulation alone (0.577 ± 0.081 mV), the combined stimulation strategy could induce stronger EMG amplitude of MG (1.426 ± 0.365 mV) after spinal cord injury (*p* < 0.01). The combined application of nINS effectively decreased the EES-induced motor threshold of MG (from 0.237 ± 0.001 mA to 0.166 ± 0.028 mA, *p* < 0.001). Additionally, the pulse width (PW) of nINS had a slight impact on the regulation of muscle activity. The EMG amplitude of MG only increased by ~70% (from 3.978 ± 0.240 mV to 6.753 ± 0.263 mV) when the PW increased by 10-fold (from 1 to 10 ms).

**Conclusion:**

The study demonstrates the feasibility of epidural combined electrical and optical stimulation for highly specific regulation of muscle activity after SCI, and provides a new strategy for improving motor dysfunction caused by SCI.

## Introduction

Spinal cord injury (SCI) refers to the structural and functional damage of the spinal cord, caused by various reasons. Due to the poor regenerative capacity of the central nervous system, SCI usually results in permanent and irreversible damage to neural pathways (Young, 2014). Under normal circumstances, motor commands from the cerebral cortex can provide nonspecific drive and maintain excitability of the spinal cord neural network. However, the interruption of descending pathways abolishes descending motor commands, resulting in the loss of drive and decrease in the excitability of spinal cord circuits. This phenomenon leads to the incomplete or complete sensory and motor dysfunction below the level of injury, resulting in symptoms, such as limb paralysis, pain, sensory disturbances, and urethral dysfunction (Estores, 2003).

The treatment of SCI is one of the pressing challenges in the field of biomedicine. Various biological therapies have been currently used for the treatment of SCI, such as anti-inflammatory drug treatment (Xu et al., 1992), infusion of neurotrophic factors (Sharma, 2007), transplantation of stem cells (Tsuji et al., 2010), and implantation of biomaterials (Duan et al., 2015; Yang et al., 2015; Rao et al., 2018; Siddiqui et al., 2021; Zhao et al., 2022). However, most of these therapeutic approaches are still in the clinical trial stage, and their clinical efficacy for SCI remains to be determined. Epidural electrical stimulation (EES) is an engineering strategy for treating SCI; it places stimulating electrodes on the surface of the spinal dura to activate the local neural network of the spinal cord by stimulating the dorsal root nerves with electric pulses, thereby promoting recovery of motor function in patients (Lavrov et al., 2008). EES was first used to treat chronic pain (Shealy et al., 1967) and later found to effectively induce muscles innervated by target nerves. Wenger et al. (2016) combined 5-hydroxytryptamine agonists, EES, and rehabilitation training in spinally transected rats and promoted rhythmic weight-bearing movements during stimulation. Clinically, Gill et al. (2018) reported that patients with complete loss of motor function with T6 incomplete SCI could stand and walk on a treadmill after 43 weeks of rehabilitation training combined with EES. Rowald et al. (2022) developed specific stimulation software that can support multiple activities, reproducing the activation of motor neurons for different types of movements. Three completely paralyzed patients with SCI were able to stand, walk, and swim with EES. In addition, this team developed a brain–spinal interface that successfully connected the brain with the spinal cord regions involved in walking, enabling completely paralyzed patients to achieve movements, such as standing, walking, and climbing stairs (Lorach et al., 2023). However, the stimulation parameters of EES exhibit high individual differences. Mesbah et al. (2023) reported that the length of the spinal column and spinal cord, location of the conus tip and the relationship between the spinal cord levels and vertebral levels, particularly at the lumbosacral enlargement, are variable across individuals. There is no statistically significant correlation between the length of the spinal column and the length of the spinal cord. The location of the spinal cord levels with respect to the electrode contacts varies across individuals and impacts the recruitment patterns of neurophysiological responses. These findings highlight the crucial role that the neuroanatomical characteristics of the spinal cord specific to each individual play in achieving maximum functional benefits with spinal cord electrical stimulation. Therefore, optimizing stimulation parameters can help improve the rehabilitation potential of EES. Rejc et al. (2015) and Angeli et al. (2023) reported that targeted selection of stimulation parameters can greatly improve the recovery of motor function in patients after epidural electrical stimulation therapy and they further enhance autonomous control of stepping by combining cervical percutaneous and lumbar sacral epidural stimulation (Angeli and Gerasimenko, 2023). Harkema et al. (2011) reported that locomotor-like patterns could be activated when stimulation parameters were optimized for stepping.

EES has remarkable advantages for clinical application due to its non-invasive nature on neural tissues (Young, 2015) and it has become an effective means to treat functional disorders after SCI. However, EES also has some limitations. First, high-intensity electric pulses can create uncontrollable electrical fields due to current diffusion, resulting in activation of nontarget neurons and reduced selectivity of EES. Second, high-intensity EES could introduce considerable charges, which may cause damage to adjacent neural tissues, and it is prone to muscle fatigue and abnormal contraction. Furthermore, electric stimulation can generate stimulating artifacts, which could potentially overwhelm evoked neural signals (Cayce et al., 2015). One strategy to overcome these limitations is to lower the intensity of electric pulses. However, how to maintain the expected stimulation effect while lowering EES intensity requires further investigation.

Recent studies showed that optical neural stimulation is an effective alternative to the conventional electrical stimulation. Infrared neural stimulation (INS) is a potential technique that offers promising solutions to the limitations of EES. Wells et al. (2007) showed that INS activates neural tissue on the basis of photothermal effect. After infrared light irradiation on nerve tissue, the tissue water absorbs the infrared light, and the photothermal effect converts laser energy into heat, causing an increase in the temperature of the neural tissue. The resulting generated transient thermal gradient changes the cell membrane capacitance and activates ion channels, ultimately leading to action potentials generation. INS has several attractive advantages compared to EES. INS is a noncontact stimulation without stimulation artifacts in recorded signals (Cayce et al., 2015). INS irradiates the target tissue through optical fibers or probes to avoid direct contact between the stimulator and tissues. INS has high spatial selectivity (Wells et al., 2005; Cayce et al., 2011), and the stimulation effect is primarily limited to irradiation points with diameters typically ranging from 100 to 400 μm (Chernov and Roe, 2014). INS has been shown to activate rat sciatic nerves with 2,120 nm laser light (Wells et al., 2005), somatosensory cortex with 1,875 nm laser light (Cayce et al., 2011), visual cortex with 850 nm laser light (Wu et al., 2013), auditory nerves with 2,120 nm laser light (Izzo et al., 2006), and vestibular nerves with 1,470 nm laser light (Bec et al., 2012). These studies have established the safety of using INS to stimulate neural tissues, with an average temperature change between 2°C and 10°C, without any tissue damage (Wells et al., 2007; Yoo et al., 2013; Zhou et al., 2022).

A previous study (Wells et al., 2007) has suggested that the optimal wavelength for INS stimulation is primarily within the mid-infrared range (1,800–2,000 nm). However, in the present study, an 808 nm near-infrared light was used, which is different from prior research. Earlier studies were mainly based on the instantaneous rise in temperature induced by INS to promote neuronal activity, hence the use of higher absorption coefficient wavelengths. However, in the present study, EES and nINS were used, hence the lower energy requirements for light stimulation. Thus, a near-infrared light with a smaller absorption coefficient was utilized. The advantages of this approach include two aspects: (i) a smaller absorption coefficient means greater penetration depth for near-infrared light, enabling its thermal effects to reach deeper spinal regions and provide effective stimulation for neurons distributed in deeper areas (Zhou et al., 2022); (ii) lower absorption rates means broader safe exposure range, significantly reducing the likelihood of thermal damage (Cayce et al., 2015).

Although the absorption coefficient of near-infrared light is smaller than that of mid-infrared light, it can still activate neural tissue. Wang et al. (2017) successfully induced auditory brainstem responses in deaf guinea pigs by stimulating their auditory neurons with 810 nm nINS. Yoo et al. (2013) reported that using 20–40 mW and 808 nm nINS could induce neuronal behavior changes in the deep brain of rats. Furthermore, research by Coventry et al. (2020) demonstrated that composite nerve action potentials could be generated in rat sciatic nerves when stimulated with 700–900 nm infrared light. Cayce et al. (2015) reported the first successful application of INS to activate human nerves. The team successfully induced motor action potentials of spinal dorsal root ganglion sensory axons by utilizing INS. These studies proved that INS-mediated neuronal activity is not restricted to specific wavelengths. Shapiro et al. (2012) revealed that heat gradients induced by INS promote the depolarization of lipid bilayer membranes by altering membrane capacitance through thermal conduction, independent of particular ion channels. Therefore, theoretically, nINS can also activate neural tissue.

On the basis of INS, a stimulation strategy that combines EES with near-INS (nINS) was proposed. First, the relationship among the EES parameters, the location of EES, and the movement responses of different muscles in the hindlimbs of SCI rats were investigated to select appropriate stimulation parameters and locations. Subsequently, on the basis of determining the EES parameters and locations, the effects of EES + nINS combined stimulation on the activation of lower limb muscles were investigated, and the regulatory effects of nINS power and pulse width (PW) on lower limb muscle activities during the combined stimulation were revealed. This study aimed to explore the feasibility of using EES + nINS to regulate muscle activity after SCI. The combination of EES and nINS was hypothesized to effectively and specifically activate the target muscles while reducing the required stimulation intensity of EES.

## Materials and methods

### Animals and experimental design

Ten female SPF-level Sprague–Dawley rats (weighing 230–290 g, 8–10 weeks old) were used in this experiment. The animals were randomly divided into EES group and nINS + EES group (*n* = 5 each). The EES group received SCI surgery, electrode implantation, and only EES. This group was used to investigate the effects of different EES sites on muscle activation and identify specific stimulation sites that activate target muscles. After the stimulation site was determined, the relationship between EES frequency/intensity and muscle activation was further explored. Meanwhile, the nINS + EES group received SCI surgery, electrode implantation, and nINS + EES. nINS + EES was then applied on the identified stimulation specific stimulation sites that activate target muscles and the relationship between nINS power/PW and muscle activation was investigated.

All rats were housed in a clean incubator with a constant temperature of 20°C, a humidity of 50%, and a 12-h light/dark cycle. They were also given access to sufficient food and water. Surgeries were performed under sterile conditions.

### SCI surgeries

**Figure 1:**
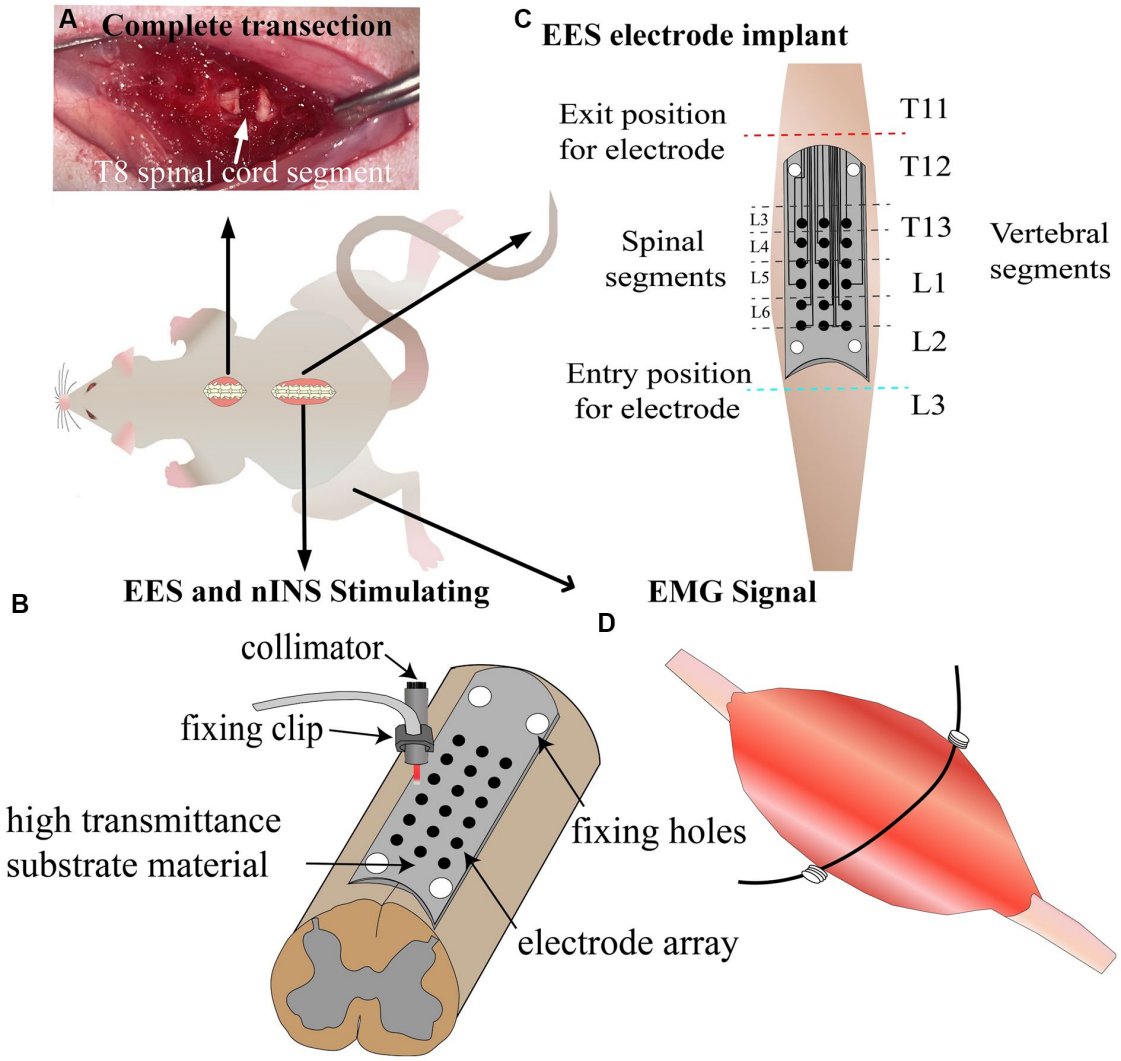
Schematic of experimental design. **(A)** Complete transection lesion at T8 spinal cord segment. **(B)** Schematic diagram of EES and nINS. **(C)** Implantation of stimulation electrodes 1 week post-injury. **(D)** Implantation of recording electrodes to record lower limb muscle EMG signals induced by stimulation. EES, epidural electrical stimulation; nINS, near-infrared neural stimulation; EMG, electromyography signals.

The rats were anesthetized with intraperitoneal injection of pentobarbital sodium (30 mg/kg), and they received skin preparation and disinfection of the surgical area. The skin was incised at the T8 vertebral level on the back, and the muscles and spinous processes in this area were removed. Partial laminectomy was performed at the T8 vertebrae to expose the spinal cord, and T8 spinal cord complete transection was performed using surgical scissors (Figure 1A). Hemostatic sponges were inserted into the transection gap, and the muscles and skin were sutured after adequate hemostasis. After the surgery, manual pressure on the bladder was applied two times daily to assist urination, and gentamicin (8 mg/kg) was injected for 7 consecutive days following SCI to prevent infection.

### Implantation of stimulation electrodes

In this study, flexible patch electrodes (Kedou Brain-Machine Technology Co., Ltd.) were implanted between the dura mater and lamina to achieve EES. Meanwhile, nINS was applied using a fixed collimator to irradiate the outer side of the electrical stimulation site. The electrode was manufactured with flexible circuit board technique, with width and length of 3 and 20 mm, respectively. The electrode substrate was made of polyimide film with an upper and lower thickness of 50 μm and good light transmittance. The electrode included a circular metal contact array of 3 × 6 and fixing clips, with pure platinum material for the electrode contacts, each with a diameter of 0.4 mm and horizontal and vertical distances of 0.7 and 2.5 mm, respectively, between neighboring contact centers. The electrode was stably fixed between the spinal cord and lamina due to its reserved attachment holes. As shown in Figure 1B, nINS was delivered in pulse mode using an 808 nm laser (MDL-808-5 W) through a collimator, which was fixed at a distance of 1 mm from the surface of the dura mater using a fixed bracket, and the fiber diameter of the collimator was 400 um.

For the EES group, electrode implantation surgery was performed 7 days after the SCI surgery. The rats were anesthetized with an intraperitoneal injection of pentobarbital sodium (30 mg/kg), and they received skin preparation and disinfection of the surgical area. A skin incision was made at the T11-L3 vertebrae to remove the muscles and spinous processes. Partial laminectomy was performed at the T11 and L3 vertebral segments to expose the spinal cord at the entry and exit sites of the stimulation electrode. The stimulating surface of the electrode contacted the dura mater, and the insulating surface contacted the vertebrae. The electrode was passed under the spinous processes and above the dura mater of the remaining vertebrae between the partial laminectomy sites with 4.0 surgical sutures. During the implantation surgery, an optical microscope was used to observe and adjust the electrode position, ensuring that the stimulation array of the electrode was located at the midline and bilateral sides (0.7 mm away from the midline) of the T13-L2 vertebral segments, and the corresponding spinal cord segments for each stimulation site are shown in Figure 1C. The flexible stimulation electrode was tightly attached to the surface of the dura mater through natural compression of the lamina. In order to further ensure that the electrode position did not deviate from its intended location, an electrophysiological test was conducted. EES (1 Hz, 0.2 mA) was applied to a series of midline sites in the electrode array, and the EMG response intensities of bilateral muscles were observed. Adjustments were made to the electrode position to avoid generating a unilateral EMG response when stimulating the midline sites. Subsequently, EES testing was carried out. A series of electrical stimulation parameters (0.2–0.6 mA and 20–60 Hz) were examined and compared, and the stimulation site was determined.

For the nINS + EES group, the procedure for electrode implantation surgery and anesthesia was identical to that described above. The corresponding vertebrae were opened to expose the spinal cord for nINS. Subsequently, nINS + EES testing was performed. A range of optical stimulation parameters (power: 0.0–2.0 W; pulse width: 0–10 ms) were studied, analyzed, and compared.

Throughout the electrode implantation and subsequent testing process, the animals were anesthetized. A heating pad was used throughout the entire experiment to maintain the body temperature of the rats at around 36°C.

### Stimulation protocol

Multichannel electrical stimulation pulses were generated using National Instruments, and LabVIEW software was used to control the stimulation parameters. EES was performed with cathodal current stimulation, and the anode was placed subcutaneously on the right shoulder of the rats. The order of the various testing was the same for each rat.

When investigating the spatial specificity/selectivity of EES, the stimulation current and frequency were 0.3 mA and 40 Hz, respectively. When investigating the effect of EES current intensity, EES was applied unilaterally to the L3 and L6 spinal segments, using burst pulse with 40 Hz, 0.2 ms PW, 0.2 s burst duration, and 1 s burst interval, with current ranging from 0.2 mA to 0.6 mA and a step size of 0.1 mA. When investigating the effect of EES frequency, EES was applied unilaterally to the L3 and L6 spinal segments by using a burst pulse with 0.2 mA, 0.2 ms PW, 0.2 s burst duration, and 1 s burst interval, with frequency ranging from 20 Hz to 60 Hz and a step size of 10 Hz. The rats rested for 2 min when changing the stimulation parameters and sites.

When investigating the combined effects of EES and nINS on muscle activation, EES consisted of isolated pulses with 1 Hz and 0.3 mA. When investigating the regulation of nINS power, the output power range of nINS was 0–2 W with a step size of 0.2 W and a PW of 5 ms. When studying the regulation of nINS PW, the output PW range of nINS was 1–10 ms with a step size of 1 ms (see Supplementary Figure 1A). The output mode of nINS and EES is shown in Supplementary Figure 1B, with EES starting to deliver a pulse at the falling edge of nINS (see Supplementary Figure 1B). An alternating stimulus with a frequency of 1 Hz was applied.

### Electromyography implants

Bipolar EMG recording electrodes were implanted in the lower limb muscles to record stimulation-induced electromyographic activity. Considering the important role of medial gastrocnemius (MG) and tibialis anterior (TA) in stance and swing phases of gait, they were selected as the target muscles for observation in this study. The EMG electrodes were made using stainless-steel wires (KD-W-304/6) coated with silicone rubber. A 1 mm incision was made on the insulation to serve as the recording electrode. One end of the stainless-steel wire was inserted into a 24-gauge injection needle. After the skin was prepared, a small incision was made on it, and the stainless-steel wire was implanted into the target muscle by using an injection needle. The recording electrode was sutured onto the muscle, with the electrode wire wound at the entrance to provide stress relief. The ground wire was inserted subcutaneously on the right shoulder with a 1 cm incision at the distal end. The insulated outer layer of the proximal end was removed and connected to the AD Instruments bioamplifier to record muscle activity (Figure 1D).

### Recording of electromyography

The EMG sampling frequency was set at 40 kHz, and AD Instruments bioamplifier was connected to PC with multichannel LabChart software for real-time recording and display of the stimulation and EMG signals. For each stimulation protocol of each rat, the trials were repeated at least three times. For each trail, measurements were repeated at least five times (at least five responses were recorded). For each trial, the order of stimulus parameters is random. The averaged EMG signal was calculated to minimize variability. The preprocessing of the EMG signal (40 kHz) included amplification, baseline drift removal, and band-pass filtering with a 10–500 Hz Butterworth filter. When determining the motor threshold (MT) of EES, the testing current began at 0.05 mA and was increased in steps of 0.01 mA until overt joint movement was observed. The current intensity at this point was defined as the MT of this muscle. The ratio of the EMG amplitude of the target muscle to that of the corresponding antagonistic muscle on the same side in L3 and L6 segments was defined as the specific activation intensity of the target muscle at that location. A larger specific activation intensity indicates better activation specificity of the target muscle. Matlab 2017 was used to write programs to perform EMG denoising, filtering, and calculation by using the peak-to-peak EMG amplitude, muscle-specific activation intensity, and motion threshold as evaluation indicators.

### Statistical analysis

Statistical analysis was performed using SPSS 24.0. Kolmogorov–Smirnov test was used to test data normality, and Levene’s test for homogeneity of variance was conducted to determine the homogeneity of variance. After verification, the data in this experiment were found to meet the normal distribution and homogeneity of variance criteria. Therefore, one-way ANOVA with Bonferroni (homogeneous variance) multiple-comparison correction was used to detect differences in muscle response under different EES intensity, frequency, nINS power, and PW. Pearson’s correlation analysis was used to calculate the correlation among EES intensity, frequency, and different lower limb muscle activities. A significance level of *p* < 0.05 was considered statistically significant. Data are presented as mean ± SD.

## Results

### Specific activation regions of muscles

**Figure 2:**
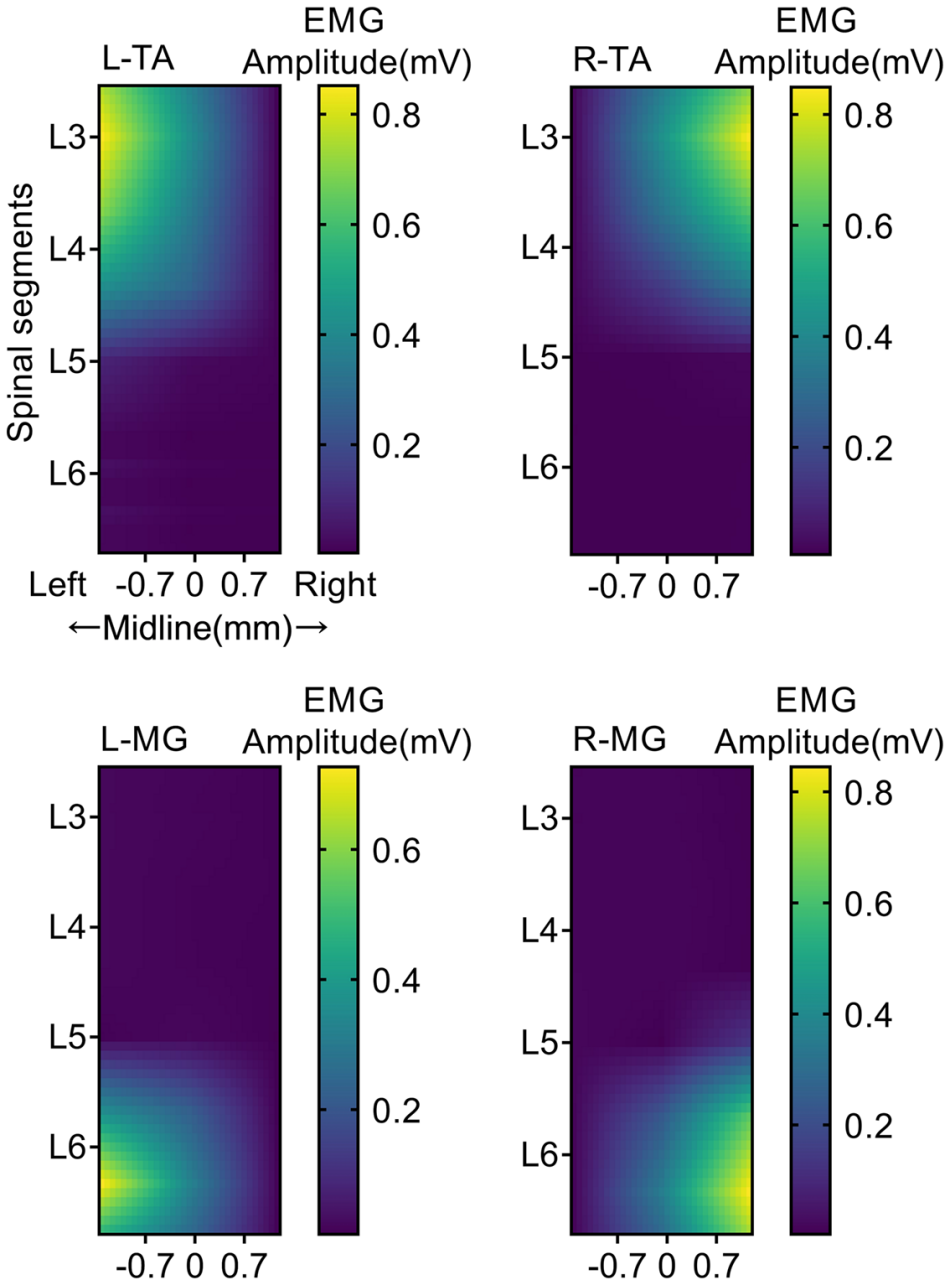
Muscle activity heatmaps activated by EES. EMG response heatmap of TA and MG of both hindlimb under different EES positions. Each panel represents the averaged EMG responses for a specific muscle when independently stimulating the 18 different electrode locations. The trials were repeated four times, and measurements in each trial were repeated seven times. The colored bar indicates the intensity of the stimulus response. L, Left; R, Right; TA, tibialis anterior; MG, medial gastrocnemius; EMG, electromyographic signal. *N* = 5 animals for each analysis.

The location of EES on the spinal cord can greatly affect its effect on muscle activation. As shown in Figure 2, each panel represents the average EMG responses for a specific muscle when independently stimulating the 18 different electrode locations. Detailed results are reported in Supplementary Tables 1–4. When EES was applied to the L3 spinal segment, activation of the flexor muscle (TA) reached its maximum. When EES was applied to the L6 spinal segment, activation of the extensor muscle (MG) reached its maximum. For lateralized activation, the EMG amplitude of the left (ipsilateral to the stimulation) TA (0.850 ± 0.002 mV; Supplementary Table 1) was significantly higher than that of the right (contralateral to the stimulation) TA (0.018 ± 0.002 mV; Supplementary Table 2) when EES was applied on the left side (*p* < 0.001). However, when EES was applied on the right side, the EMG amplitude of the right (ipsilateral to the stimulation) TA (0.848 ± 0.140 mV) was significantly higher than that of the left (contralateral to the stimulation) TA (0.008 ± 0.002 mV, *p* < 0.001). When EES was applied at the L3 midline, the EMG amplitudes of bilateral tibialis anterior muscles were 0.424 ± 0.024 and 0.427 ± 0.008 mV, indicating both muscles were activated. However, their amplitudes were lower than the muscle responses of the left TA (0.850 ± 0.002 mV) and right TA (0.848 ± 0.140 mV) under lateralized stimulation (*p* < 0.05). Four sites of the bilateral L3 and L6 spinal segments were chosen as the optimal specificity stimulation sites for TA and MG on the stimulation side to achieve specific activation.

### Effects of stimulation current on muscle activation

**Figure 3:**
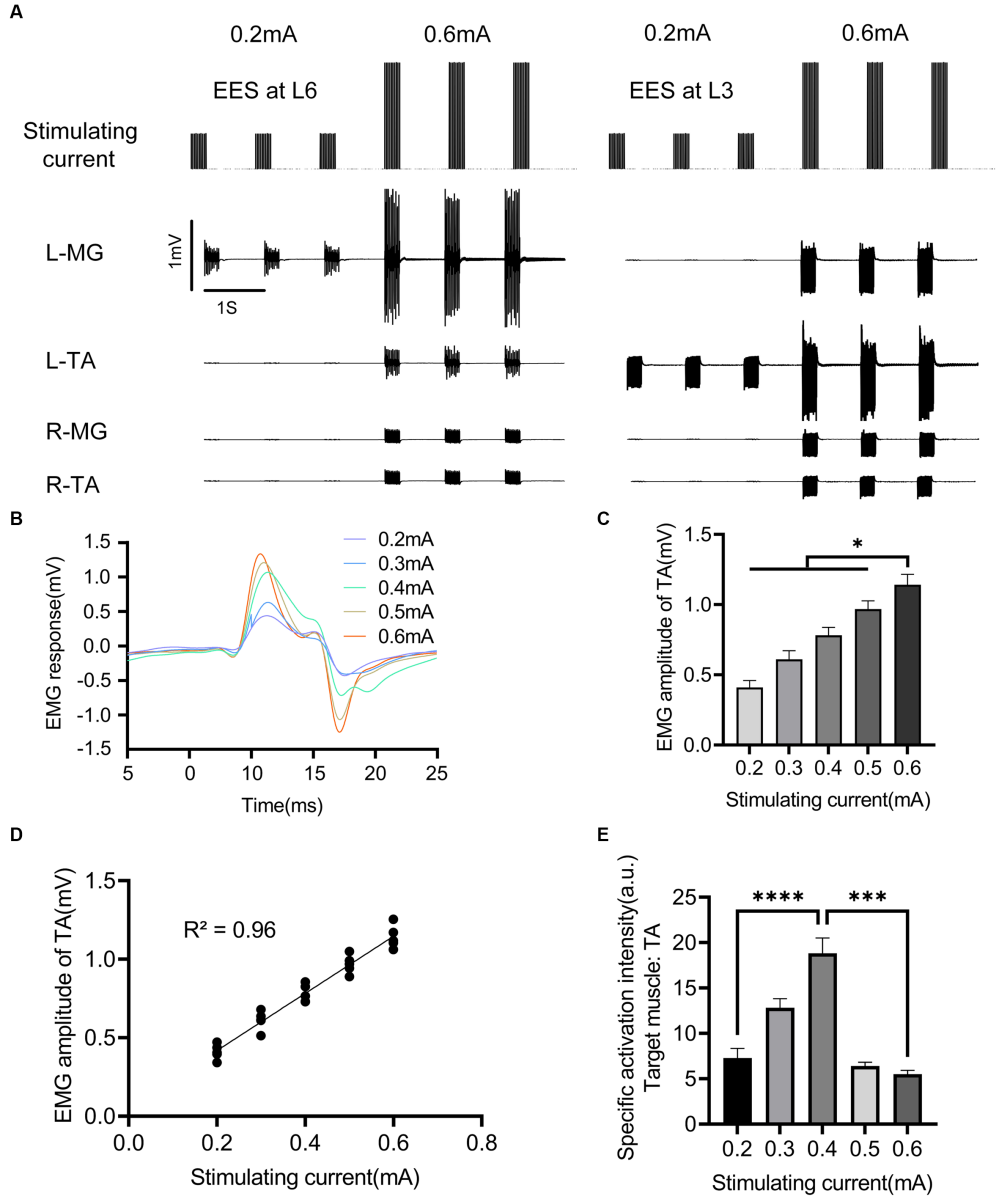
Effects of EES intensity on evoked muscle activity. **(A)** EMG signals of main muscles under different EES intensity acting on L3/L6 spinal cord segment. **(B)** EMG activity of left TA under different stimulation intensity. **(C)** Comparison of EMG amplitudes of left TA under different stimulus intensity. **(D)** Correlation between the activation intensity of left TA and stimulation intensity. **(E)** Comparison of specific activation intensity of left TA under different stimulation intensity. EES, epidural electrical stimulation; L, left; R, right; TA, tibialis anterior; MG, medial gastrocnemius; EMG, electromyographic signal. Data were presented as the mean ± SD. ^*^p < 0.05, ^***^p < 0.001, ^****^p < 0.0001 by ANOVA with Bonferroni correction or Pearson’s correlation analysis. The trials were repeated three times, and measurements in each trial were repeated six times. *N* = 5 animals for each analysis.

At the determined EES location, the effect of EES current intensity on muscle activation was investigated (Figure 3). The electrode sites located at the left-side edge of the L3 and L6 spinal cord segments were used to apply stimulation. The results showed that low-intensity EES could not induce muscle movement responses. With the increase in current intensity, the target muscle (TA/MG) was specifically activated by EES (Figure 3A). With EES at the left L3 spinal cord segment as an example, the EMG amplitude of the target muscle (left TA, ipsilateral to the stimulation) increased with the increase in stimulation current (Figure 3B). The muscle response induced by high-intensity EES was significantly stronger than that induced by low-intensity EES (Figures 3C, *P* < 0.01). When the stimulation current was 0.6 mA, the EMG amplitude of TA (1.142 ± 0.074 mV) was significantly stronger than the response when the stimulation current was 0.2 mA (0.410 ± 0.049 mV, *p* < 0.0001). In addition, the EMG amplitude of the target muscle (left TA) was positively correlated with the stimulation’s current intensity (*p* < 0.0001; Figure 3D). When the stimulation intensity reached 0.6 mA, the antagonistic muscle (left MG) and contralateral muscle (right TA) of the target muscle were activated (Figure 3A). Therefore, although the stronger the stimulation current, the stronger the activation intensity of the target muscle, the specificity of the target muscle gradually decreased (*p* < 0.005, Figure 3E).

### Effects of stimulation frequency on response muscle activity

**Figure 4:**
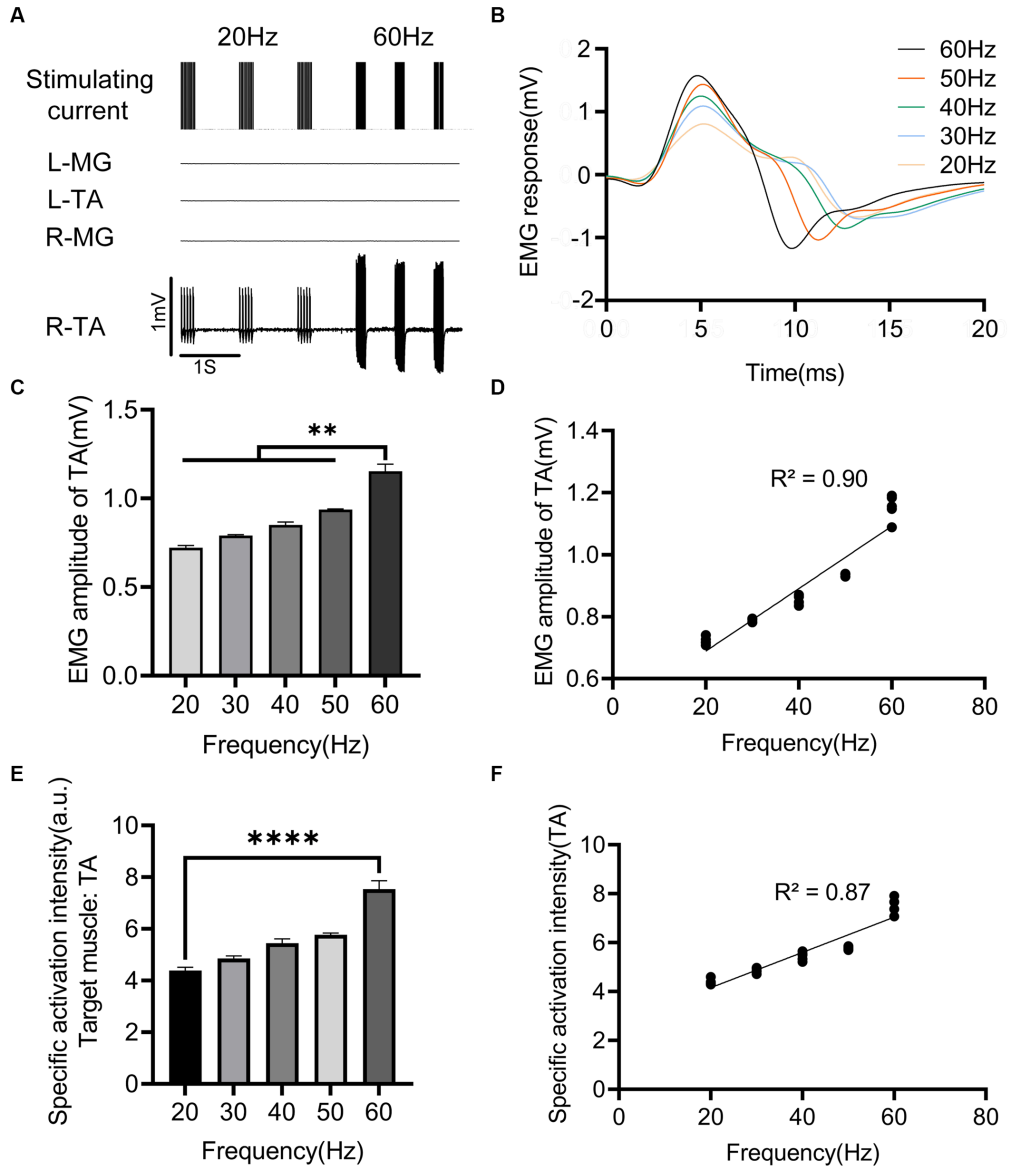
Effects of EES frequency on evoked muscle activity. **(A)** EMG signals of main muscles at different EES frequency acting on the right side of L3 spinal cord segment. **(B)** EMG responses of right TA at different stimulation frequency. **(C)** Comparison of EMG amplitudes of right TA under different stimulation frequency. **(D)** Correlation between EMG amplitude of right TA and different stimulation frequency. **(E)** Comparison of specific activation intensity of right TA under different stimulation frequency. **(F)** Correlation between the specific activation intensity of TA on the right side and different stimulation frequency. L, left; R, right; TA, tibialis anterior; MG, medial gastrocnemius; EMG, electromyographic signal. Data were presented as the mean ± SD. ^**^p < 0.01, ^****^p < 0.0001 by ANOVA with Bonferroni correction or Pearson’s correlation analysis. The trials were repeated three times, and measurements in each trial were repeated six times. *N* = 5 animals for each analysis.

The regulatory effect of EES frequency on muscle activity is shown in Figure 4. When the stimulation frequency was less than 20 Hz, the lower limb muscles exhibited intermittent contraction. When the stimulation frequency was greater than 20 Hz, higher stimulation frequencies shortened the response interval, resulting in sustained muscle contraction of the target muscle. The EMG amplitude of the right (ipsilateral to the stimulation) TA increased with increasing stimulation frequency (Figures 4A–C), and the two were positively correlated (*p* < 0.0001, Figure 4D). The regulation capability of stimulation frequency on muscle activity was weak. When the stimulation frequency increased from 20 to 60 Hz, the EMG amplitude of the target muscle (TA) only increased from 0.721 ± 0.012 mV to 1.150 ± 0.040 mV (Figure 4C). A notable detail that with the increase in stimulation frequency, the nontarget muscles did not show significant activation (Figure 4A). Thus, the specificity of the target muscle (TA) slowly increased in a linearly correlated manner (*p* < 0.0001; Figures 4E, F).

### Modulation of nINS power on muscle activation

**Figure 5:**
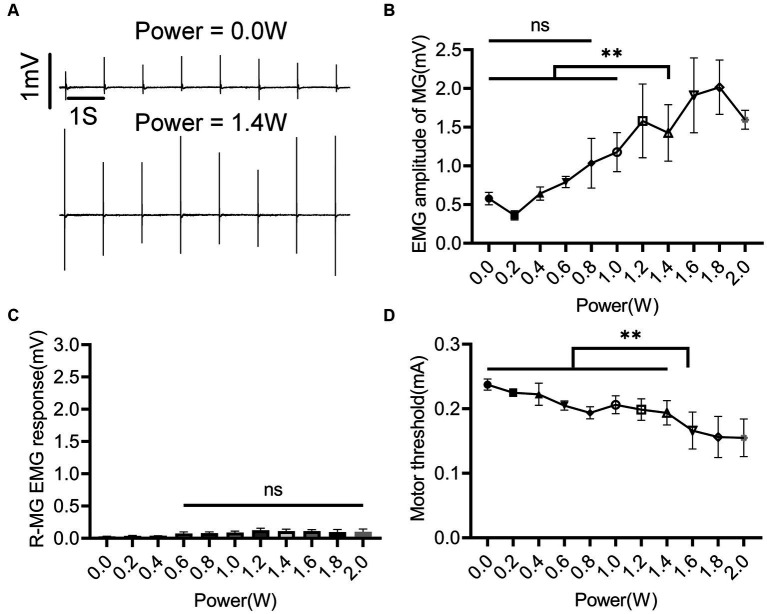
Regulatory effect of nINS power on left MG during left L6 segment stimulation with EES + nINS. **(A)** EMG response of left MG (target muscle) under EES only (0 W) and EES + nINS (1.4 W). **(B)** Comparison of EMG amplitudes of left MG under different power nINS effects. **(C)** Changes in EMG amplitude of right MG (contralateral muscle) under different nINS power effects. **(D)** MT change trend of left MG under different nINS powers. EMG, electromyographic signal; MG, medial gastrocnemius; R, right. Data were presented as the mean ± SD. ns, none significant; ^**^p < 0.01 by ANOVA with Bonferroni correction. The trials were repeated three times, and measurements in each trial were repeated five times. *N* = 5 animals for each analysis.

After the EES protocol was determined, the effect of nINS output power on muscle activity was studied. EES + nINS were applied to the left L6 spinal segment, with the target muscle being the left MG. The stimulation frequency of nINS was set at 1 Hz to avoid thermal accumulation from high-frequency exposure. As shown in Figure 5, when the nINS power was 0 (i.e., no nINS stimulation was applied), EES could not activate the left (ipsilateral to the stimulation) MG (Figure 5A). Similarly, when the output power of nINS was low (nINS power < 1 W), the joint stimulation with EES + nINS did not significantly enhance the stimulus response of MG. However, when the nINS stimulation power exceeded 1 W, the EMG amplitude of the left MG (target muscle) was significantly enhanced (*p* < 0.01), and a clear EMG response in the left MG was observed (Figure 5B). Moreover, changes in nINS power did not induce EMG responses in the ipsilateral antagonist muscle or contralateral muscle (Figure 5C). When the nINS power reached 1.6 W, the MT of the left MG significantly decreased (Figure 5D). The MT of the left MG stimulated only by EES was 0.237 ± 0.001 mA, whereas that stimulated jointly by EES + nINS (power of 1.6 W) significantly decreased to 0.166 ± 0.028 mA (*p* = 0.0005).

### Modulation of nINS PW on muscle activation

**Figure 6:**
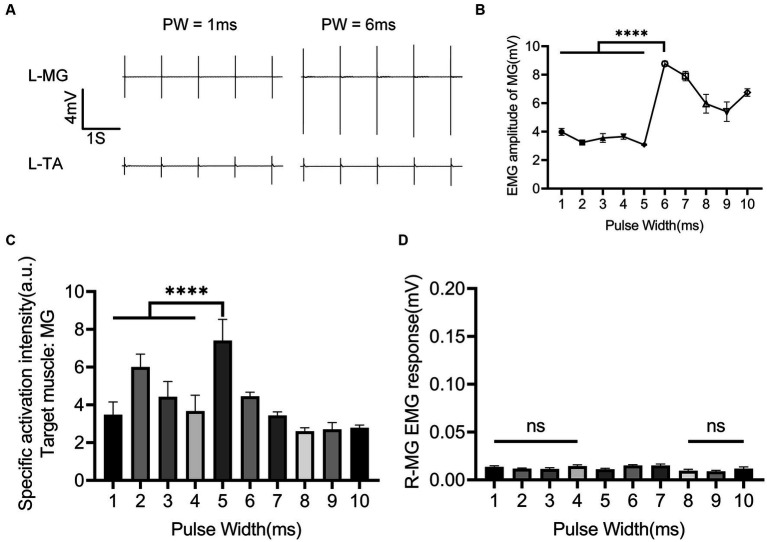
Regulatory effect of nINS pulse width on left MG when EES + nINS stimulates the left L6 segment. **(A)** EMG response of left MG (target muscle) and TA under combined stimulation of PW = 1 ms and PW = 6 ms. **(B)** Motion response of left MG (target muscle) under different pulse widths of nINS. **(C)** Specific activation intensity of left MG (target muscle) under different pulse widths of nINS. **(D)** Motor response of right MG (contralateral muscle) under different pulse widths of nINS. EMG, electromyographic signal; MG, medial gastrocnemius; R, right. Data were presented as the mean ± SD. ns, none significant; ^****^p < 0.0001 by ANOVA with Bonferroni correction. The trials were repeated three times, and measurements in each trial were repeated five times. *N* = 5 animals for each analysis.

The role of nINS PW in the combined EES + nINS stimulation is illustrated in Figure 6. The effect of changes in nINS PW on muscle activity was relatively weak (Figure 6A). When PW ≤ 5 ms, the EMG amplitude of the left (ipsilateral to the stimulation) MG (target muscle) remained essentially stable (Figure 6B). There is no significant change with PW changing from 1 to 5 ms (3.978 ± 0.240 mV to 3.080 ± 0.046 mV, *p* = 0.178). Compared with the muscle response of the target muscle during the combined stimulation at PW = 1 ms (3.978 ± 0.240 mV), the EMG amplitude of the target muscle significantly increased (8.775 ± 0.178 mA, *p* < 0.0001) when PW = 6 ms. The EMG amplitude of left MG at PW = 10 ms (6.753 ± 0.263 mV) was only about 70% higher than that at PW = 1 ms (3.978 ± 0.240 mV, *p* < 0.0001) and 12% higher than that at PW = 8 ms (5.961 ± 0.656 mV, *p* = 0.0574). In terms of specific activation of the target muscle, the best specificity was achieved at PW = 5 ms (7.405 ± 1.126; Figure 6C), which was significantly higher than that at PW = 1 ms (3.475 ± 0.679 mV, *p* < 0.0001) and PW = 6 ms (4.451 ± 0.217 mV, *p* < 0.0001). Additionally, increasing the nINS PW did not activate the contralateral muscle, nor did it induce strong activation of the ipsilateral antagonist muscle (Figure 6D).

## Discussion

The relationship between the stimulation location, current intensity, and frequency of EES and the muscle activity of the lower limb MG and TA in SCI rats was explored, and the appropriate stimulation parameters (inducing specifically tonic contractions of target muscles) and locations for EES were determined. On the basis of the determined EES protocol, the regulatory roles of nINS power and PW on muscle activity were established by combining EES with nINS. The results demonstrated that EES + nINS stimulation could specifically activate target muscles, and the required EES intensity to reach MT was significantly lower than when using EES alone.

Single technological approaches have limitations in therapeutic applications for SCI. Although EES can effectively activate target muscles, a high current intensity of the stimulation may reduce the specificity of activation. In this study, the increase in EES current led to the activation of the target muscle’s antagonist and contralateral muscles, and this finding could be partly attributed to charge diffusion caused by high current input (Wells et al., 2005). The simultaneous activity of paired antagonist muscles disrupts the normal sequence of muscle activation during movement, resulting in low-quality gait movements. Therefore, lowering EES intensity while activating the target muscle is important for regulating the orderly activity of different muscles after SCI to recover motor function.

INS offers the advantages of being noncontact and having high spatial resolution (Wells et al., 2005; Cayce et al., 2011; Chernov and Roe, 2014). Previous research on peripheral nerves has reported that INS can enhance neuronal responses to electrical stimulation, mainly by creating temperature gradients in the target area through transient light stimulation and depolarizing neurons to a certain extent, which is beneficial for neuronal excitation induced by electrical stimulation (Cayce et al., 2015). Previous studies have shown that excessive frequency of INS can cause thermal damage to tissues (Wells et al., 2007; Thompson et al., 2014). Therefore, although we chose 40 Hz in our exploration of EES, a reduced stimulus frequency was executed during EES + nINS combined stimulation to avoid potential thermal damage. INS is known to alter the cell membrane potential by generating instantaneous thermal gradients (Wells et al., 2007) and therefore produces depolarizing currents (Shapiro et al., 2012). It is expected that when INS acts on the spinal cord, depolarizing currents will increase the excitability of the group-Ia and group-II afferent nerves to EES (Moraud et al., 2016). Based on this principle, nINS and EES are set to 1 Hz pulse alternating stimulation. The superimposed currents generated by INS and EES may lead to better muscle activation than EES alone. In the present study, EES was combined with nINS, providing preliminary evidence for the feasibility of using this combination in regulating muscle activity after SCI and establishing a foundation for the development of combined strategies.

This study also found that the power of nINS plays a more significant role in muscle activation than PW. Furthermore, the effect of nINS power seems to have a threshold, and only when the applied power intensity reached this threshold (in this study, nINS power = 1 W) that the excitatory effect of EES + nINS on spinal neurons becomes evident. This phenomenon is consistent with the mechanism of INS activation of neurons mentioned earlier, that is, the thermal effect can only generate a sufficiently large temperature gradient at the stimulation location when the nINS power surpasses a specific threshold. Stimulating the spinal cord with nINS alone at the discovered threshold failed to produce any noticeable activation, proving that the combined strategy utilizes the effects of EES and nINS to induce neuronal excitation. However, the relationship among the nINS power threshold found in this study, the EES parameters used in the current experiment and its trend with the changing EES parameters, remains to be further investigated.

Although the effect of nINS PW on muscle activity is not as prominent as its power, the specificity of muscle activity remains satisfactory even with a wide range of PW variations. This finding suggested that fine-tuning muscle activation can be achieved by adjusting the PW.

However, this study has some limitations. Firstly, the mechanism of neural tissue activation by EES + nINS remains to be clarified. Although the combined strategy utilizes the effects of EES and nINS, the mode of interaction between the two requires further elucidation. Secondly, the safety ranges of EES, nINS, and the combined stimulation need to be further confirmed to determine whether a reasonable combination of EES and nINS parameters can further reduce the damage caused to spinal tissue by the applied stimulation. Thirdly, the improvement of motor function may require a multi-site stimulation paradigm. However, only a single-site EES + nINS protocol was explored in the current study. The effectiveness and reliability of multi-site combined stimulation need to be established in future studies. Fourthly, excessive current intensity of EES may lead to charge accumulation, while prolonged or excessive INS power may result in heat accumulation, which can cause irreversible tissue damage to the nerves near the stimulation site. Finally, due to the small sample size in this study, the results need to be further validated in a larger cohort. To overcome these limitations, some potential strategies are worth exploring: (i) reducing the size of the stimulation electrode array and the contact area between the spinal cord and the electrode after identifying the stimulation site; (ii) using an implantable fiber optic instead of a collimator to provide nINS under minimally invasive conditions; (iii) exploring the effects of nINS with different wavelengths and clarifying the processes of charge accumulation and heat accumulation under different nINS + EES parameters.

In this study, the role of EES + nINS combined stimulation in regulating the muscle activity of lower limb MG and TA after SCI was investigated. The findings demonstrated that epidural combined electrical and optical stimulation could significantly reduce the current intensity required for muscle activation with EES alone. At the same current intensity, the combined electrical and optical stimulation not only maintains high-specificity activation of target muscles but also enhances the muscle activation. The results confirmed the feasibility of using epidural combined electrical and optical stimulation to regulate muscle activity after SCI, laying the foundation for the development and optimization of subsequent strategies.

## Data availability statement

The raw data supporting the conclusions of this article will be made available by the authors, without undue reservation.

## Ethics statement

The animal study was approved by the Institutional Experimental Animal Ethics Committee of Beihang University (approval: BM20230028). The study was conducted in accordance with the local legislation and institutional requirements.

## Author contributions

X-JG: Formal Analysis, Investigation, Methodology, Software, Writing – original draft. ZZ: Methodology, Writing – original draft, Investigation. J-QC: Data curation, Investigation, Writing – original draft, Formal Analysis, Methodology, Validation. L-WH: Methodology, Writing – original draft, Data curation, Formal Analysis, Visualization. W-NS: Visualization, Writing – original draft, Formal Analysis, Investigation. TF: Writing – original draft, Formal Analysis, Methodology. CZ: Funding acquisition, Project administration, Software, Supervision, Visualization, Writing – review & editing. MX: Project administration, Writing – review & editing, Conceptualization, Resources, Supervision. J-SR: Conceptualization, Writing – review & editing, Funding acquisition, Project administration, Resources, Supervision, Validation.
