# Supplementary material for: Epidural combined optical and electrical stimulation induces high-specificity activation of target muscles in spinal cord injured rats
Source: Front Neurosci. 2023 Nov 10;17:1282558. doi: 10.3389/fnins.2023.1282558 (PMC10667474; doi:10.3389/fnins.2023.1282558)
Supplement: Supplementary file 1 [file Data_Sheet_1.docx]

Supplementary Material

Epidural combined optical and electrical stimulation induces high-specificity activation of target muscles in spinal cord injured rats

Xiao-Jun Guo^1,*^, Ziyi Zhao^2,*^, Jia-Qi Chang^3,*^

First Author*

*** Correspondence:**

Jia-Sheng Rao,

email:raojschina@126.com, Phone:+86-15801130200

Meng Xu,

email:drxm301@163.com, Phone:+86-13501175839

Can Zhao,

email:zhaocan05@163.com, Phone:+86-15010993562

# Supplementary Figures and Tables

## Supplementary Figures


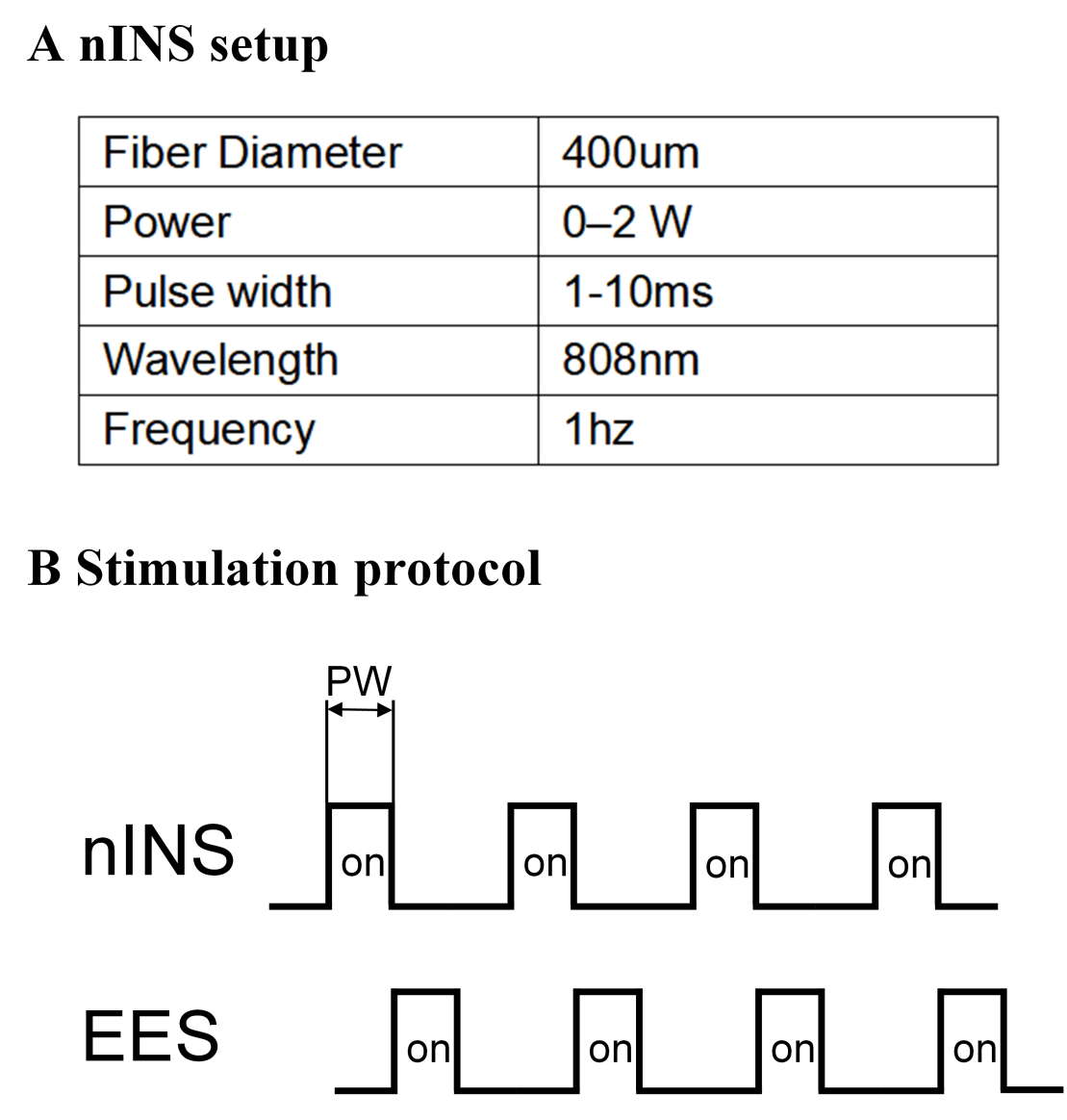


**Supplementary Figure 1.** The nINS setup and stimulation protocol. (A) The parameters setup for nINS. (B) Stimution protocol fo EES and nINS.

## Supplementary Tables

**Supplementary Table 1. Averaged EMG response of left TA (mV)**

| Vertical\Lateral | Left (-0.7 mm) | Midline (0.0 mm) | Right (+0.7 mm) |
| --- | --- | --- | --- |
| 1 | 0.850 ± 0.002 | 0.424 ± 0.024 | 0.008 ± 0.002 |
| 2 | 0.699 ± 0.010 | 0.354 ± 0.006 | 0.007 ± 0.001 |
| 3 | 0.425 ± 0.007 | 0.276 ± 0.003 | 0.008 ± 0.001 |
| 4 | 0.017 ± 0.001 | 0.019 ± 0.002 | 0.008 ± 0.001 |
| 5 | 0.017 ± 0.001 | 0.003 ± 0.001 | 0.004 ± 0.001 |
| 6 | 0.014 ± 0.001 | 0.001 ± 0.001 | 0.003 ± 0.001 |

**Note:**  Averaged EMG responses for left TA when independently stimulating the 18 different electrode locations. TA, tibialis anterior; EMG, electromyographic signal. Data are presented as mean ± SD.

**Supplementary Table 2. Averaged EMG response of right TA (mV)**

| Vertical\Lateral | Left (-0.7 mm) | Midline (0.0 mm) | Right (+0.7 mm) |
| --- | --- | --- | --- |
| 1 | 0.018 ± 0.002 | 0.427 ± 0.008 | 0.848 ± 0.140 |
| 2 | 0.017 ± 0.001 | 0.328 ± 0.009 | 0.623 ± 0.011 |
| 3 | 0.018 ± 0.002 | 0.154 ± 0.015 | 0.362 ± 0.005 |
| 4 | 0.008 ± 0.001 | 0.006 ± 0.002 | 0.020 ± 0.001 |
| 5 | 0.006 ± 0.001 | 0.007 ± 0.001 | 0.008 ± 0.001 |
| 6 | 0.007 ± 0.001 | 0.007 ± 0.001 | 0.008 ± 0.002 |

**Note:**  Averaged EMG responses for right TA when independently stimulating the 18 different electrode locations. TA, tibialis anterior; EMG, electromyographic signal. Data are presented as mean ± SD.

**Supplementary Table 3. Averaged EMG response of left MG (mV)**

| Vertical\Lateral | Left (-0.7 mm) | Midline (0.0 mm) | Right (+0.7 mm) |
| --- | --- | --- | --- |
| 1 | 0.019 ± 0.001 | 0.016 ± 0.001 | 0.011 ± 0.001 |
| 2 | 0.019 ± 0.001 | 0.013 ± 0.001 | 0.011 ± 0.001 |
| 3 | 0.021 ± 0.001 | 0.013 ± 0.001 | 0.010 ± 0.001 |
| 4 | 0.134 ± 0.012 | 0.018 ± 0.001 | 0.008 ± 0.001 |
| 5 | 0.357 ± 0.004 | 0.225 ± 0.005 | 0.011 ± 0.001 |
| 6 | 0.722 ± 0.005 | 0.331 ± 0.004 | 0.012 ± 0.001 |

**Note:**  Averaged EMG responses for left MG when independently stimulating the 18 different electrode locations. MG, medial gastrocnemius; EMG, electromyographic signal. Data are presented as mean ± SD.

**Supplementary Table 4. Averaged EMG response of right MG (mV)**

| Vertical\Lateral | Left (-0.7 mm) | Midline (0.0 mm) | Right (+0.7 mm) |
| --- | --- | --- | --- |
| 1 | 0.009 ± 0.001 | 0.011 ± 0.001 | 0.004 ± 0.001 |
| 2 | 0.011 ± 0.001 | 0.011 ± 0.001 | 0.004 ± 0.001 |
| 3 | 0.013 ± 0.001 | 0.010 ± 0.003 | 0.005 ± 0.003 |
| 4 | 0.014 ± 0.001 | 0.004 ± 0.001 | 0.136 ± 0.005 |
| 5 | 0.013 ± 0.001 | 0.238 ± 0.001 | 0.645 ± 0.003 |
| 6 | 0.013 ± 0.001 | 0.038 ± 0.051 | 0.852 ± 0.013 |

**Note:**  Averaged EMG responses for right MG when independently stimulating the 18 different electrode locations. MG, medial gastrocnemius; EMG, electromyographic signal. Data are presented as mean ± SD.
